# Supplementary material for: Polyketide synthase gene clusters in the ericoid mycorrhizal fungus Oidiodendron maius: genome-wide prediction, expression profiling, and functional characterization of knockout mutants
Source: Front Plant Sci. 2026 Sep 16;17:1924430. doi: 10.3389/fpls.2026.1924430 (PMC13623903; doi:10.3389/fpls.2026.1924430)
Supplement: Supplementary file 1 [file SupplementaryFile1.zip › Supplementary Materials/Supplementary Folder 1.zip!/Oidma1_AssemblyScaffolds_Repeatmasked/knownclusterblast/cluster6/jgi.p_Oidma1_48208_mibig_hits.html]

| MIBiG Protein | Description | MIBiG Cluster | MiBiG Product | % ID | % Coverage | BLAST Score | E-value |
| --- | --- | --- | --- | --- | --- | --- | --- |
| Bcin12g06400.1 |  | BGC0000631 | Botrydial\_biosynthetic\_gene\_cluster | 41 | 96.1315280464 | 404 | 1.3e-112 |
| BAV32149.1 | cytochrome\_P450\_monooxygenase | BGC0001373 | Sordarin\_biosynthetic\_gene\_cluster | 41 | 100.386847195 | 363 | 3.4e-100 |
| BAV32152.1 | cytochrome\_P450\_monooxygenase | BGC0001373 | Sordarin\_biosynthetic\_gene\_cluster | 35 | 101.740812379 | 332 | 6.4e-91 |
| AIA58898.1 | putative\_cytochrome\_P450 | BGC0001141 | Brefeldin\_biosynthetic\_gene\_cluster | 37 | 100.967117988 | 304 | 2.4e-82 |
| AAK33073.1 | cytochrome\_P450 | BGC0001278 | Nivalenol\_/\_deoxynivalenol\_/\_3-acetyldeoxynivalenol\_/ | 36 | 97.0986460348 | 303 | 4.2e-82 |
| AAK33083.1 | putative\_cytochrome\_P450 | BGC0001277 | Nivalenol\_/\_deoxynivalenol\_/\_3-acetyldeoxynivalenol\_/ | 36 | 97.8723404255 | 292 | 1.3e-78 |
| AAK53584.1 | trichodiene\_oxygenase | BGC0000931 | Trichothecene\_biosynthetic\_gene\_cluster | 36 | 97.8723404255 | 291 | 1.6e-78 |
| AAK53577.1 | trichodiene\_oxygenase | BGC0000930 | Trichothecene\_biosynthetic\_gene\_cluster | 35 | 97.6789168279 | 287 | 4e-77 |
| AIA58895.1 | putative\_cytochrome\_P450 | BGC0001141 | Brefeldin\_biosynthetic\_gene\_cluster | 33 | 100.386847195 | 285 | 1.2e-76 |
| AIA58896.1 | putative\_cytochrome\_P450 | BGC0001141 | Brefeldin\_biosynthetic\_gene\_cluster | 35 | 97.6789168279 | 284 | 3.4e-76 |
| ADM79460.1 | P450\_protein | BGC0001266 | Grayanic\_acid\_biosynthetic\_gene\_cluster | 32 | 107.156673114 | 272 | 7.9e-73 |
| AAC49958.1 | cytochrome\_P450 | BGC0001279\_c2 | Deoxysambucinol\_/\_sambucinol\_/\_roridin\_E\_biosynthetic\_gene\_cluster | 33 | 96.5183752418 | 260 | 4.1e-69 |
| AAS89997.1 | CypA | BGC0000007 | Aflatoxin\_biosynthetic\_gene\_cluster | 31 | 92.4564796905 | 231 | 2e-60 |
| AAS90045.1 | CypA | BGC0000009 | Aflatoxin\_biosynthetic\_gene\_cluster | 30 | 97.8723404255 | 220 | 3.6e-57 |
| Bcin12g06380.1 |  | BGC0000631 | Botrydial\_biosynthetic\_gene\_cluster | 30 | 93.6170212766 | 216 | 6.7e-56 |
| EHA22197.1 | hypothetical\_protein | BGC0000170 | Yanuthone\_D\_biosynthetic\_gene\_cluster | 32 | 96.5183752418 | 209 | 8.2e-54 |
| AGA37280.1 | P450\_monooxygenase | BGC0000819 | Paraherquamide\_biosynthetic\_gene\_cluster | 28 | 94.9709864603 | 180 | 5.3e-45 |
| BAJ09784.1 | P450 | BGC0000146 | Solanapyrone\_biosynthetic\_gene\_cluster | 28 | 88.0077369439 | 172 | 1.1e-42 |
| ABV57820.1 | cytochrome\_P450\_monooxygenase | BGC0001267 | Lysergic\_acid\_/\_elymoclavine\_biosynthetic\_gene\_cluster | 28 | 87.8143133462 | 159 | 1.3e-38 |
| BAD83682.1 | cytochrome\_P-450 | BGC0000012 | Alternapyrone\_biosynthetic\_gene\_cluster | 26 | 90.3288201161 | 146 | 6.5e-35 |
| BAD29972.1 | P450\_monooxygenase\_1 | BGC0000676 | Aphidicolin\_biosynthetic\_gene\_cluster | 25 | 88.2011605416 | 140 | 6.1e-33 |
| EED17999.1 | benzoate\_4-monooxygenase\_cytochrome\_P450,\_putative | BGC0000154 | Stipitatic\_acid\_biosynthetic\_gene\_cluster | 25 | 72.3404255319 | 94 | 5e-19 |
| ctg1\_orf000000 |  | BGC0000685 | Brassicicene\_C\_biosynthetic\_gene\_cluster | 55 | 20.8897485493 | 123 | 7.7e-28 |
| AAC49196.1 | putative\_p450\_monooxygenase | BGC0000152 | Sterigmatocystin\_biosynthetic\_gene\_cluster | 25 | 90.1353965184 | 123 | 1e-27 |
| ACH72905.1 | AflG | BGC0000011 | Aflatoxin/sterigmatocystin\_biosynthetic\_gene\_cluster | 25 | 91.6827852998 | 118 | 2.5e-26 |
| AGC83578.1 | P450\_monooxygenase | BGC0000818 | Notoamide\_biosynthetic\_gene\_cluster | 24 | 96.9052224371 | 108 | 3.4e-23 |
| ADM34140.1 | P450 | BGC0001084 | Notoamide\_/\_stephacidin\_biosynthetic\_gene\_cluster | 25 | 91.6827852998 | 102 | 1.4e-21 |
| ADZ25011.1 | cytochrome\_P450\_hydroxylase | BGC0000380 | Leupyrrin\_biosynthetic\_gene\_cluster | 35 | 33.8491295938 | 89 | 9.5e-18 |
| ADE22337.1 | monooxygenase | BGC0000065 | Galbonolides\_biosynthetic\_gene\_cluster | 32 | 34.4294003868 | 77 | 4.9e-14 |
| AEE83590.1 | cytochrome\_P450,\_family\_705,\_subfamily\_A,\_polypeptide\_4 | BGC0001313 | Arabidiol\_/\_Baruol\_biosynthetic\_gene\_cluster. | 32 | 32.6885880077 | 73 | 9.2e-13 |
| EER93095.1 | hypothetical\_protein | BGC0000798 | Dhurrin\_biosynthetic\_gene\_cluster | 25 | 30.1740812379 | 60 | 6.2e-09 |
